# SUPPORTING DIVERSE FAMILY CAREGIVERS

**DOI:** 10.1093/geroni/igac059.1201

**Published:** 2022-12-20

**Authors:** Pamela Nadash

**Affiliations:** University of Massachusetts Boston, Boston, Massachusetts, United States

## Abstract

The RAISE Family Caregiving Advisory Council, created under the Recognize, Assist, Include, Support, and Engage (RAISE) Family Caregivers Act (2018) has been tasked to support the Secretary of Health and Human Services in developing a national family caregiving strategy. This presentation reports on research commissioned to support the activities of the Council, which aimed to engage a broad range of stakeholders in identifying concrete strategies to carry out the Goals identified as critical to supporting family caregivers. One priority was to engage with organizations representing the diversity of family caregivers, including groups working with Blacks, Indigenous people, Asian-Americans, Pacific Islanders, and other people of color, along with groups such as faith organizations that work with under-resourced communities. Respondents had much to say regarding mechanisms for ensuring access to services and supports among diverse family caregivers, most notably identifying support for “community ambassadors” as key, as well as targeted awareness campaigns.

